# Therapeutic potential of gastro-gastric fistulas created via lumen-apposing metal stents for nutritional deficiencies after roux-en-y gastric bypass

**DOI:** 10.1055/a-2713-0016

**Published:** 2025-10-16

**Authors:** Kambiz Kadkhodayan, Azhar Hussain, Abdullah Abassi, Saurabh Chandan, Sagar Pathak, Gustavo Bello Vincentelli, Natalie Cosgrove, Mustafa A Arain, Maham Hayat, Deepanshu Jain, Artur Viana, Mohamad Khaled Almujarkesh, Tareq Alsaleh, Magda Elamin, Nihal Ijaz Khan, Dennis Yang, Shayan Irani, Muhammad Khalid Hasan

**Affiliations:** 1 440172 Center for Interventional Endoscopy, AdventHealth Orlando, Orlando, United States; 2 473255 Medicine, Ameer-ud-Din Medical College of PGMI, Lahore, Pakistan; 3 558924 Bariatric Surgery, AdventHealth Central Florida, Orlando, United States; 4 440172 Gastroenterology, AdventHealth Orlando, Orlando, United States; 5 7289 Gastroenterology and Hepatology, Virginia Mason Medical Center, Seattle, United States

**Keywords:** Endoscopic ultrasonography, Intervention EUS, Endoscopy Lower GI Tract, Stenting

## Abstract

**Background and study aims:**

Roux-en-Y gastric bypass (RYGB) is an effective long-term weight loss operation with improvements in metabolic diseases. Nutritional deficiencies, however, are highly prevalent despite supplementation, largely due to exclusion of the proximal small bowel. In RYGB patients who require pancreaticobiliary access, the EUS-directed transgastric ERCP (EDGE) procedure provides a stable gastro-gastric (GG) fistula using a lumen-apposing metal stent (LAMS). The metabolic and nutritional effects of temporary food diversion remain unknown.

**Methods:**

We conducted a review of 60 consecutive RYGB patients from two tertiary centers who underwent EDGE. Nutritional and metabolic parameters were assessed before LAMS placement and after removal.

**Results:**

Mean age was 63.2 ± 11.05 years; 23% were male. Significant improvements were observed in serum hemoglobin (mean difference (MD) 1.1 g/dL; *P* = 0.004), vitamin B12 levels (MD 204.4 pg/mL; *P* = 0.021), iron (MD 57.9 mcg/dL; *P* = 0.017), albumin (MD 0.4 g/dL; *P* = 0.013), and magnesium levels (MD 0.24 mg/dL; *P* = 0.016). In addition, serum folate (MD 2.2 μg/mL; *P* = 0.873), and ferritin levels (MD 315.5 μg/mL; *P* = 0.335), showed improvement trends, but these did not reach statistical significance. No significant changes were observed in total body weight, body mass index, serum glucose, hemoglobin A1c, serum triglycerides, low-density lipoprotein, or high-density lipoprotein ( *P* > 0.05 for all).

**Conclusions:**

Temporary partial-reversal of RYGB using a LAMS improves key nutritional parameters without compromising metabolic benefits of RYGB. These findings may support a therapeutic role for iatrogenic GG fistulas as a minimally invasive option for RYGB patients with refractory nutritional deficiencies.

## Introduction

Roux-en-Y gastric bypass (RYGB) is among the most widely performed operations, known for its effectiveness in achieving sustained weight loss, as well as improvement in metabolic conditions such as type 2 diabetes mellitus (T2DM), dyslipidemia, hypertension, and cardiovascular disease. These benefits are primarily attributed to exclusion of the gastric remnant, duodenum and proximal jejunum, which leads to changes in hormonal responses, gut microbiota, and nutrition absorption. Although RYGB-induced malabsorption has several benefits, it may also predispose patients to multiple nutritional deficiencies that are often challenging to manage medically 1 2 3 4 .

Post-RYGB nutritional deficiencies are well-documented in literature and occur due to the bypassing of duodenum and proximal jejunum, which are critical sites for the action of digestive enzymes and nutrient absorption. Despite oral supplementation, deficiency in fat-soluble vitamins (vitamin A, D, E and K) and water-soluble vitamins (B12, folic acid, thiamine, Vit C) affect as many as 60% of patients within 2 years of RYGB 5 . In addition, deficiencies in key minerals such as iron, calcium, magnesium, zinc, copper, and magnesium are also common because they are primarily absorbed in bypassed segments of small intestine 5 6 7 . Severe protein-calorie malnutrition is prevalent in up to 4.7% of patients 1 to 2 years after surgery 8 9 . This is largely due to delayed mixing of digestive enzymes, which is essential for breakdown and absorption of proteins, carbohydrates, and fats 10 .

Nutritional deficiencies in RYGB patients are difficult to supplement orally, and often un-preventable with standard multivitamin supplementation 11 . It is estimated that the cost of multivitamin supplementation alone, per-RYGB patient per year, in the Netherlands is €1,224 12 . As a result, clinical presentations of dietary deficiency, such as refractory anemia, protein malnutrition, and osteoporosis, are highly prevalent and result in recurrent hospitalization and increased healthcare utilization 10 13 .

In recent years, endoscopic ultrasound (EUS)-directed transgastric endoscopic retrograde cholangiopancreatography (EDGE) has provided a stable gastro-gastric (GG) or jejunal-gastric (JG) fistula via use of a lumen-apposing metal stent (LAMS) for patients with RYGB who require pancreaticobiliary procedures 14 15 16 . While in-situ, the LAMS serves as a shunt that partially reverses the malabsorptive bypass in RYGB by diverting a portion of ingested food into the excluded stomach. Although there is ample literature that supports safety, efficacy, and utility of EDGE for pancreaticobiliary disorders, metabolic and nutritional effects of this temporary rerouting of food into the excluded stomach and small bowel remain largely unknown.

We hypothesized that partial shunting of ingested food and its reexposure to native digestive enzymes and the absorptive surface of bypassed small bowel might help correct nutritional deficiencies without adversely affecting metabolic benefits of RYGB. This study evaluated short-term nutritional and metabolic effects of temporary, partial and reversible rerouting of food via iatrogenic GG/JG fistulas.

## Patients and methods

This was a retrospective review of prospectively collected data that included consecutive adult patients (aged ≥18 years) with RYGB who underwent EDGE and subsequent removal of LAMS between 2022 and 2025 at two large tertiary hospitals. Patients with irreversible coagulopathy, international normalized ratio > 1.5, platelet count < 50,000 mm ^3^ , inability to stop anticoagulants or antiplatelet medication, pregnancy, inability to consent, or contraindications to sedation were excluded.

All EDGE procedures were performed under general anesthesia. A curvilinear echoendoscope was used, and under endosonographic guidance, the excluded stomach was accessed with a 19G needle and expanded with a solution of water-soluble radiocontrast and water. This was followed by free-hand placement of a cautery-enhanced LAMS (15 mm or 20 mm) to connect the gastric pouch or afferent jejunal limb and excluded stomach. All patients underwent a pancreatic or biliary procedure either during the index procedure (same-session ERCP) or during a different procedure (staged ERCP). The LAMS was removed via endoscopy after completion of the second procedure, usually after 6 to 8 weeks.

Demographic, clinical, and laboratory data collection occurred at the time of LAMS placement or at preadmission testing (up to 2 months prior to LAMS placement), time of LAMS removal, and at 6-month follow-up (0, 3, and 6 months).

Statistical analysis was conducted using SPSS version 25 (IBM Corporation, Armonk, New York, United States). Paired *t* -test was used to evaluate changes between data points obtained at LAMS placement and LAMS removal. Linear curve regression with Pearson correlational analysis was used to evaluate the linear relationship between the various nutritional parameters and LAMS indwell time. The Holm-Bonferroni step-down approach was applied to determine risk of false positives and adjusted *P* values. Effect sizes were determined using Cohen’s d. Nutritional deficiency was defined as any laboratory value falling below the established normal reference range. Statistical significance was defined as a two-sided *P* < 0.05.

## Results

A total of 60 patients with RYGB who underwent an EDGE procedure were included in the analysis. Mean age was 63.2 ± 11.05 years [male 14 (23.3%), female 46 (76.7%)]. Common indications were choledocholithiasis (n = 46), bile leak (n = 5), biliary stricture (n = 5), pancreatic necrosis with common bile duct dilation (n = 1), pancreatic mass (n = 2), and duodenal perforation with biliary dilatation (n = 1). Median time since RYGB was 18 years, (range 3–38 years). Median LAMS indwell time was 44.5 days (range 17–123 days).

**Fig. 1:**
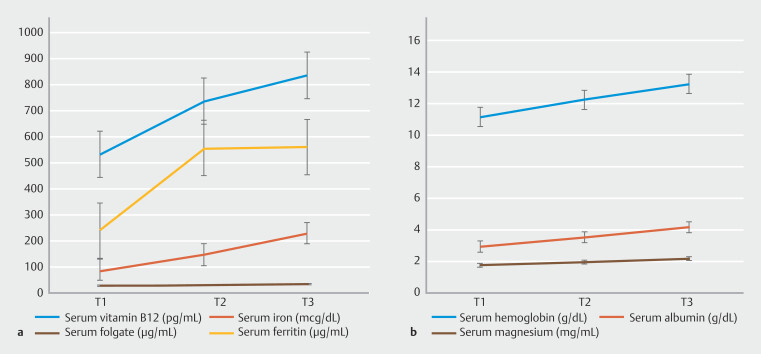
**a** , **b** Trends in various nutritional parameters of the study on the multi-line graph over time before and after LAMS placement.

When comparing pre- and post-EDGE nutritional parameters, paired *t* -test analysis revealed a statistically significant improvement in serum hemoglobin (mean difference 1.1 g/dL; *P* = 0.004), serum vitamin B12 levels (mean difference 204.4 pg/mL; *P* = 0.021), serum iron (mean difference 57.9 µg/dL; *P* = 0.017), serum albumin (mean difference 0.4 g/dL; *P* = 0.013), and serum magnesium levels (mean difference 0.24 mg/dL; *P* = 0.016). Adjusted *P* values using the Holm-Bonferroni method for serum hemoglobin, vitamin B12, serum iron, serum albumin, and serum magnesium levels were below the critical threshold of statistical significance ( *P* < 0.05), confirming the statistical significance of these findings while accounting for the increased risk of false positives. In addition, there was an improvement trend in serum folate (mean difference 2.2 μg/mL; *P* = 0.873) and serum ferritin levels (mean difference 315.5 μg/mL; *P* = 0.335), but these did not reach statistical significance ( Fig. 1 ).

**Fig. 2:**
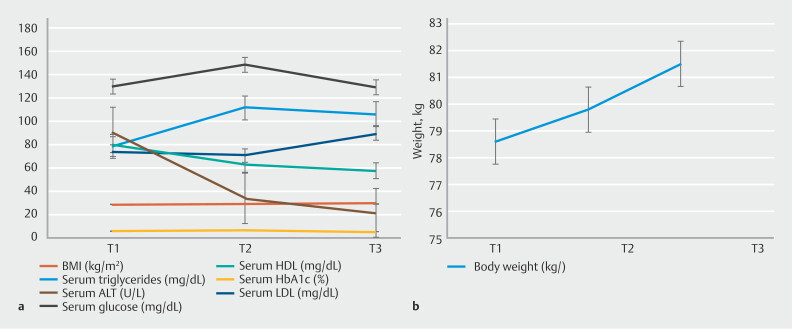
**a** , **b** Trends in various metabolic parameters and total body weight of the study population on the multi-line graph over time before and after LAMS placement.

When comparing pre- and post-EDGE metabolic parameters, there was no statistically significant difference in total body weight, body mass index (BMI), serum glucose, hemoglobin A1c, serum triglycerides, low-density lipoprotein, or high-density lipoprotein ( *P* > 0.05 for all). Interestingly, there was a statistically significant reduction in serum alanine aminotransferase (ALT) (mean difference 57.03 U/L; *P* = 0.012) ( Fig. 2 , Table 1 ).

**Table:** **Table 1** Summary of various pre- and post-EDGE nutritional and metabolic parameters distribution with mean differences.

Parameter	Normal range	Mean pre-EDGE (T1) ± SD	Mean post-EDGE (T2) ± SD	Mean difference (T2-T1) (95% Confidence Interval) ± SD	*P* value (Cohen’s *d* )
**Metabolic parameters**					
Body weight (kg)	-	78.6 ± 17.2	79.8 ± 20.1	1.1 (-3.8–1.5) ± 3.4	0.389 (0.32)
BMI (kg/m ^2^ )	18–25	29.3 ± 6.1	29.6 ± 7.2	0.3 (-1.3- 0.7) ± 3.2	0.531 (0.09)
Serum random glucose (mg/dL)	70–200	130.4 ± 40.5	149.2 ± 75.7	18.8 (-8.6–46.4) ± 78.8	0.172 (0.23)
Serum HbA1c (%)	< 5.7%	6.3 ± 1.4	6.6 ± 1.6	0.2 (-0.2–0.7) ± 0.5	0.277 (0.40)
Serum random triglycerides (mg/dL)	< 150	79.0 ± 44.2	112 ± 68.9	33.5 (-50.1–117.1) ± 55.2	0.292 (0.60)
Serum HDL (mg/dL)	> 40	80.5 ± 23.8	63.7 ± 34.3	-16.7 (-37.4–3.9) ± 12.9	0.082 (1.20)
Serum LDL (mg/dL)	< 129	74.7 ± 21.5	71.3 ± 33.8	-3.4 (-65.5–58.7) ± 39.0	0.873 (0.08)
Serum ALT (U/L)	4–51	91.6 ± 103.1	34.5 ± 46.7	-57.0 (-100.5–13.5) ± 118.5	0.012 (0.50)
**Nutritional parameters**					
Serum hemoglobin (g/dL)	Men: 13.5–17.5; Women: 12.0–15.5	11.2 ± 1.8	12.3 ± 2.0	1.1 (0.3–1.8) ± 2.0	0.004 (0.55)
Serum vitamin B12 (pg/mL)	232–1245	531.8 ± 269.3	736.2 ± 277.0	204.4 (42.0–366.6) ± 194.1	0.021 (1.05)
Serum iron (mcg/dL)	Men: 75–150; Women: 60–140	89.2 ± 43.4	147.14 ± 84.5	57.9 (14.7–100.9) ± 46.5	0.017 (1.24)
Serum albumin (g/dL)	3.2–5.5	3.0 ± 0.6	3.57 ± 0.52	0.4 (0.06–0.5) ± 0.6	0.013 (0.66)
Serum magnesium (mg/dL)	1.7–2.2	1.8 ± 0.2	2.0 ± 0.1	0.2 (0.03–0.2) ± 0.2	0.016 (1.0)
Serum folate (μg/mL)	4.8–20	28.0 ± 27.4	30.2 ± 11.3	2.2 (-147.1–142.6) ± 16.1	0.873 (0.13)
Serum ferritin (μg/mL)	11–307	239.5 ± 14.8	555.0 ± 263.0	315.5 (-2181.2- 2812.2) ± 277.8	0.335 (1.13)
ALT, alanine aminotransferase; BMI, body mass index; EDGE, endoscopic ultrasound-directed transgastric endoscopic retrograde cholangiopancreatography; HbA1c, hemoglobin A1c; HDL, high-density lipoprotein; LDL, low-density lipoprotein.					

**Fig. 3:**
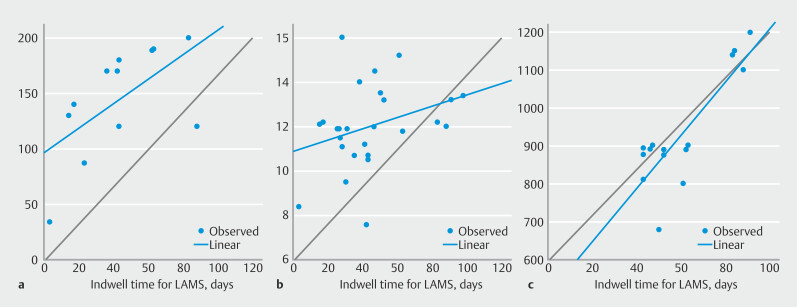
**a** Pearson correlation analysis revealing serum iron levels have strong positive correlation with indwell time of LAMS post-EDGE procedure (r = 0.997; *P* = 0.003). **b** Pearson correlation analysis revealing serum hemoglobin have moderately strong positive correlation with indwell time of LAMS post-EDGE procedure (r = 0.55; *P* = 0.045). **c** Pearson correlation analysis revealing that serum vitamin B12 levels have strong positive correlation with indwell time of LAMS post-EDGE procedure (r = 0.85; *P* = 0.013).

Pearson correlation analysis revealed that serum hemoglobin (r = 0.55; *P* = 0.045), serum iron levels (r = 0.997; *P* = 0.003), and serum vitamin B12 levels (r = 0.85; *P* = 0.013) had a moderate to strong correlation with LAMS indwell time ( Fig. 3 ).

## Discussion

**Fig. 4:**
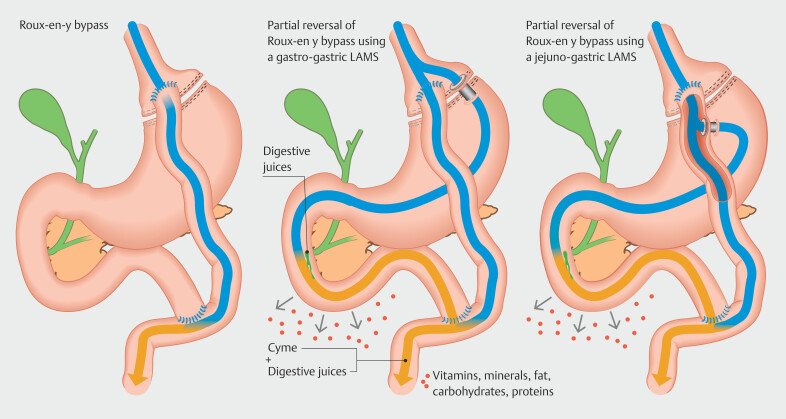
Illustration demonstrating normal post-RYGB anatomy (left) and partial reversal of RYGB via a gastro-gastric LAMS (middle) or jejunal-gastric LAMS (right). Note that after partial reversal, the nutrient rich stream of chyme is partially shunted toward the excluded stomach, duodenum, and biliopancreatic limb, where it mixes with digestive juices, thus partially restoring native physiological absorption of nutrients.

Our study demonstrates that temporary and partial reversal of RYGB anatomy via a LAMS is associated with significant improvement in nutritional parameters without significantly altering metabolic benefits of bypass. We observed statistically significant increases in serum hemoglobin, iron, vitamin B12, albumin, and magnesium, which are frequently deficient following RYGB despite oral supplements. In addition, the iatrogenic GG/JG fistula did not result in significant weight gain, change in glucose, hemoglobin A1C, lipid profile or BMI. Our findings support our hypothesis that partially restoring the gastric chyme flow toward the excluded stomach and bypassed small bowel enables more physiological digestion and absorption of nutrients and corrects underlying deficiencies that can be difficult to treat ( Fig. 4 ). In addition, our findings suggest that metabolic benefits of RYGB are preserved despite partial bypass reversal. The minor and non-significant metabolic changes may have been due to the limited indwell time of the LAMS and only partial reversal of the bypass. These findings are important, because it is inherent fear of reversing metabolic benefits of RYGB that often deters more aggressive nutritional interventions in RYGB patients, despite recurrent hospital admissions and high healthcare resource utilization.

The nutritional improvements observed in our patient cohort are consistent with the altered digestive physiology of RYGB in which exclusion of the duodenum and jejunum limits exposure of ingested nutrients to pancreatic enzyme bile and the absorptive surface of the excluded proximal small bowel, which are essential for absorption of fat-soluble vitamins (A,D,E,K), water-soluble vitamins, and micronutrients and macronutrients. By reestablishing a conduit between the gastric pouch and excluded stomach, the GG/JG fistula enables partial shunting of the gastric stream into the excluded stomach, facilitating early mixing of digestive juices, bile, and ingested nutrients, which restores normal physiological digestion and nutrient uptake in the duodenum and jejunum. In addition, improved iron and B12 absorption is likely due to restored chyme exposure to gastric acid and intrinsic factors, which are crucial for B12 absorption 17 18 . Although resolution of common bile duct obstruction is likely a major contributor to the observed ALT improvement, restoration of micronutrient homeostasis may also play a role. Vitamin E, in particular, functions as an antioxidant that prevents hepatocellular injury from lipid metabolism and choline is essential for hepatic very-low-density lipoprotein metabolism 19 20 21 . Deficiency in these nutrients is well-documented in RYGB 22 . Early reexposure of the ingested nutrients to the proximal small bowel through the GG/GJ fistula likely improves absorption and hepatocellular metabolism, although that requires further mechanistic studies.

We observed a correlation between LAMS indwell time and improvement in specific nutritional markers (hemoglobin, iron, B12). This suggests a potential dose-response effect, and may present an opportunity to personalize therapy by titrating LAMS indwell time based on severity of patient nutritional deficits. Durability of observed benefits at 6-month follow-up suggests a sustained mid-term nutritional benefit after LAMS removal, although longer-term follow-up is necessary.

From a healthcare utilization perspective, our study may carry important implications. Although also speculative, use of a temporary LAMS may reduce overall healthcare utilization and cost when compared with our current approach of managing refractory nutritional deficiencies. As an example, patients with severe post RYGB anemia may require repeated iron infusions and hospitalizations for blood transfusions or anemia workup. A minimally invasive intervention that restores endogenous nutrient absorption capacity may reduce the overall cost of managing such patients, although that requires further study.

Not all RYGB patients with nutritional deficiencies are likely to benefit from LAMS placement. Based on our experience, ideal candidates include patients with severe or refractory nutritional deficiencies despite well-documented adherence to nutritional supplementation. Another cohort of patients who are likely to benefit are patients requiring repeated hospitalization for anemia, protein-calorie malnutrition, or electrolyte imbalances. It is important to emphasize that patients with mild nutritional deficiencies that are well controlled on supplements, with poor adherence or inability to undergo repeat anesthesia for LAMS removal, or patients with poorly controlled metabolic disease may not benefit from this minimally invasive approach.

Our study has several limitations. First, it has a retrospective design that limits causal inference, and although the data were prospectively collected, the study is subject to selection bias. Second, our sample size, although the largest reported for this application, is still inadequate to detect more subtle metabolic effects and rare complications of LAMS and may not be entirely representative. Third, duration of GG fistula maintenance was short. This may not fully capture the true metabolic effects of a persistent longer-term fistulae. Finally, our cohort required pancreatic biliary intervention and included patients with medical illness including malignancy. The effects of a GG fistula on such patients may not fully reflect the performance of LAMS in otherwise healthy patients.

Despite its limitations, our study offers a novel insight into the therapeutic potential of temporary GG/JG fistulae, not as an access route for endoscopic retrograde cholangiopancreatography, but as a minimally invasive intervention for RYGB patients who suffer from refractory nutritional deficiencies. With the growing number of bariatric operations being performed globally, such applications may offer an effective, minimally invasive alternative to definitive surgical revision in management of post-bypass malnutrition.

## Conclusions

Temporary creation of an EUS -guided GG/JG fistula with use of a LAMS results in significant improvement in nutritional parameters without adversely impacting metabolic benefits of RYGB. These effects may be highly beneficial and represent a novel therapeutic option for carefully selected RYGB patients with nutritional deficiencies that are refractory to medical management.
